# Endoscopic ischemic polypectomy for small intestinal polyps in a 7‐year‐old girl with juvenile polyposis syndrome

**DOI:** 10.1002/jpr3.70188

**Published:** 2026-04-13

**Authors:** Shingo Kurasawa, Shun Watanabe, Satoshi Ukai, Ayako Furuya, Kato Sawako, Yoshiko Nakayama

**Affiliations:** ^1^ Department of Pediatrics Shinshu University School of Medicine Nagano Japan

**Keywords:** crossed‐clip strangulation method, double‐balloon enteroscopy, endoscopic treatment

## Abstract

This is the first report of endoscopic ischemic polypectomy (EIP) for small intestinal polyps in a pediatric patient with juvenile polyposis syndrome (JPS). A 7‐year‐old girl underwent double‐balloon enteroscopy, during which 17 pedunculated polyps were treated using the crossed‐clip strangulation method without complications. Ten‐month follow‐up confirmed complete clip detachment, no residual lesions, and resolution of anemia and hypoalbuminemia, and growth was satisfied. EIP, avoiding electrocautery, may theoretically reduce the risk of bleeding and perforation and represents an alternative option for managing small intestinal, pedunculated, clearly benign polyps in children with JPS.

## 1 INTRODUCTION

Juvenile polyposis syndrome (JPS) is a rare autosomal dominant hereditary disorder characterized by the development of multiple hamartomatous polyps in the stomach, small intestine, and colon. Pediatric patients may present with hematochezia, anemia, hypoalbuminemia, growth retardation, and intussusception.^1^ Endoscopic surveillance during childhood aims to reduce the risk of intussusception, and the European Society for Pediatric Gastroenterology, Hepatology and Nutrition (ESPGHAN) recommends polypectomy for polyps ≥10 mm in diameter.^2^

Patients with JPS are predisposed to polyp formation throughout life, warranting repeated endoscopic surveillance and treatment. Polyps exceeding 15 mm in diameter pose a higher risk of intussusception.^3^ Surgery is indicated if nonsurgical reduction fails or intestinal ischemia is suspected. However, postoperative adhesions may hinder deep enteroscopic access and necessitate further surgical interventions, reducing the quality of life. Double‐balloon enteroscopy (DBE) enables minimally invasive treatment, making it the preferred approach over surgery in pediatric patients.

Funayama et al.^4^ described the technique of endoscopic ischemic polypectomy (EIP) in detail in both pediatric and adult patients with Peutz–Jeghers syndrome (PJS). Subsequently, EIP was reported to be a safe and effective treatment for 269 small intestinal polyps in 22 pediatric PJS cases.^5^ To the best of our knowledge, this study is the first report of EIP performed for small intestinal polyps in a pediatric patient with JPS.

## 2 CASE REPORT

The patient was a 7‐year‐old girl with no family history of JPS and a medical history of surgical repair of a ventricular septal defect and pulmonary artery stenosis. At the age of 3, the patient was referred to our department with rectal polyp prolapse, facial edema associated with hypoalbuminemia (Albumin: 2.6 g/dL), hematochezia, anemia (Hemoglobin: 8.5 g/dL), and her height standard deviation score (SDS) indicated a growth rate delay, dropping from –0.8 to –1.5 SDS during the 9 months leading up to the onset. Gastrointestinal endoscopy revealed multiple hamartomatous polyps in the stomach, small intestine, and colon. The polyps ranged in size from 5 to 25 mm. Histological examination of resected polyps confirmed the diagnosis of juvenile polyps. Accordingly, a diagnosis of JPS was reached. Genetic testing identified a de novo pathogenic variant in the *BMPR1A* gene (NM_004329.2:c.1438 C > T:p. Arg480Trp). The patient underwent repeated endoscopic polypectomies, resulting in improvements in anemia, hypoalbuminemia, and growth. The patient underwent her first small bowel capsule endoscopy (SBCE) at 3 years of age, and small intestinal polyps <5 mm in size were detected. The second SBCE, performed at 4 years of age, revealed multiple 10 mm‐sized pedunculated polyps. The first DBE was performed, and 9 small intestinal polyps were resected.

**Figure 1:**
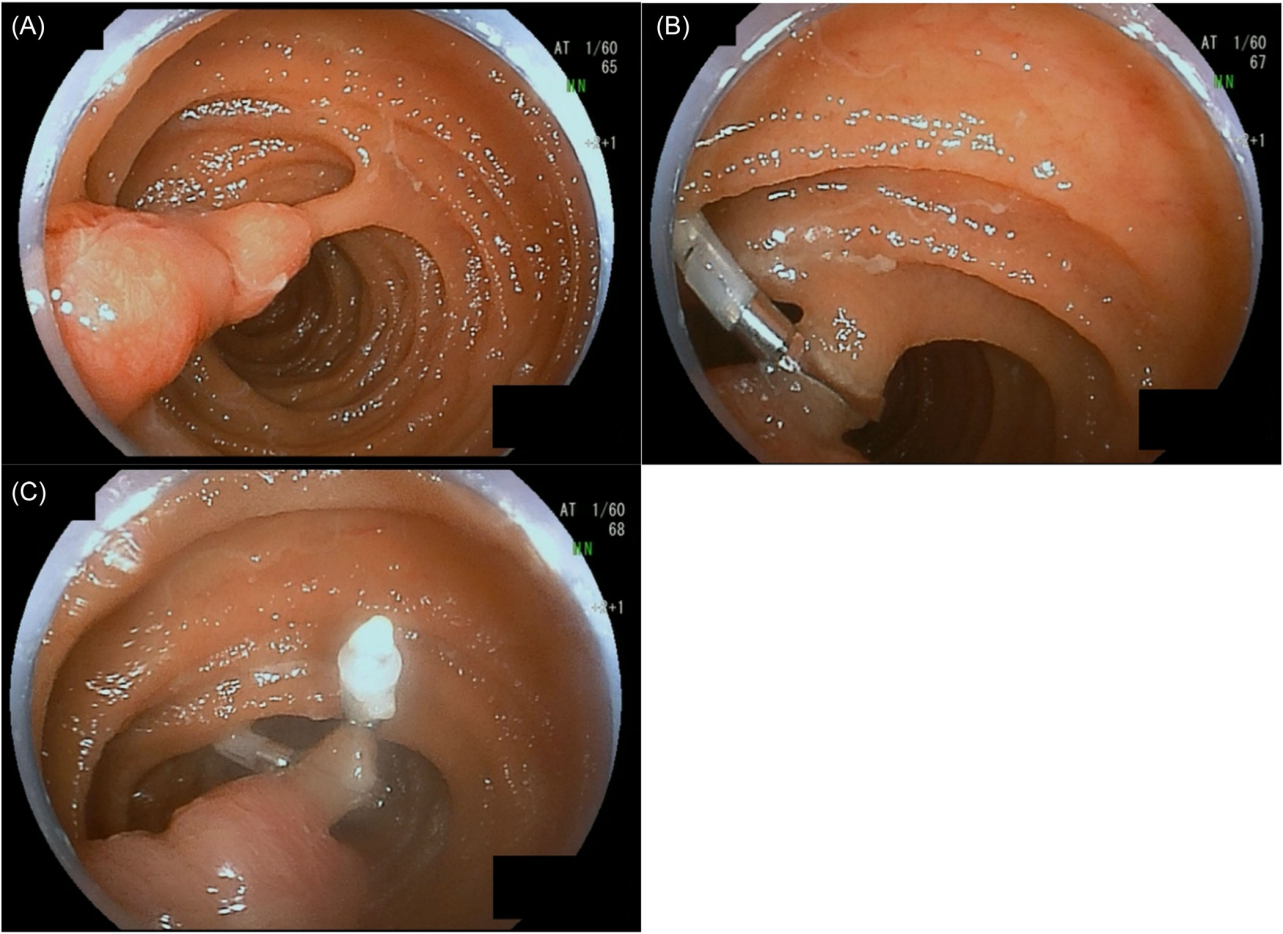
(A) Pedunculated polyps in the small intestine. (B) The first hemostatic clip was placed on the stalk of the polyp. (C) A second hemostatic clip was placed on the stalk of the polyp at a 90‐degree angle to the first clip.

At 7 years of age, the patient underwent her third SBCE for surveillance, revealing multiple small intestinal pedunculated polyps. A second DBE was performed under general anesthesia using the EN‐580T (FUJIFILM Medical Co., Ltd.). At that time, the patient's height was 116.2 cm (−1.0 SDS), and weight was 24.9 kg (−0.5 SDS). Endoscopic mucosal resection (EMR) was performed to address two nonpedunculated polyps. To treat 17 pedunculated polyps, all with a visually assessed size range of 7–15 mm, EIP was performed using the “crossed‐clip strangulation method,” in which the first hemostatic clip was deployed with the aid of a distal attachment and rotated 90°, followed by placement of a second clip crossing the first at a 90° angle (Figure 1). The procedure was completed without complications. The endoscopic treatment of small intestinal polyps required 116 min.

Ten months thereafter, DBE was performed, and pedunculated small intestinal polyps were subjected to EIP; the absence of residual clips from the previous EIP was confirmed. Subsequently, anemia and hypoalbuminemia did not recur, and growth was satisfied.

## 3 DISCUSSION

EIP induces ischemic necrosis and spontaneous detachment of polyps by clipping the base and occluding blood flow. Its efficacy and safety in treating small intestinal polyps have been reported in both adults and children with PJS.^4^, ^5^ This is the first reported case of EIP using the crossed‐clip strangulation method in a pediatric patient with JPS.

Among the potential complications of EMR and hot snare polypectomy (HSP) for small intestinal polyps, delayed perforation and bleeding are particularly concerning. The small intestine, which is highly mobile within the abdominal cavity, has a thinner wall and narrower lumen than the colon, making it more susceptible to these complications. To date, there have been no reports regarding the application of CSP for polyps in the deep small intestine. According to the Japan Gastroenterological Endoscopy Society guidelines, cold snare polypectomy (CSP) is recommended for lesions <10 mm in diameter.^6^ Based on recent reports, including those by Attard et al.^7^ and Friesen et al.,^8^ CSP is considered a safe and effective therapeutic option for polyps in children with JPS when the polyps are nonpedunculated and measure less than 10 mm in diameter. CSP has been reported to carry a low risk of delayed bleeding. In the present case, many of the polyps were pedunculated and measured greater than 10 mm. Therefore, EIP was selected. Combining CSP with EIP to obtain histologic specimens is a consideration for the future.

Currently, there are no reports analyzing complications associated with DBE‐based resection of small intestinal polyps in pediatric JPS. In PJS, the incidence rates of delayed bleeding and perforation following therapeutic DBE were reportedly 2.7% and 1.4%, respectively.^9^ Unlike EMR and HSP, EIP does not involve electrocautery, eliminating the risk of thermal injury and offering a safer alternative for small bowel lesions, especially in children. A limitation of EIP is the inability to retrieve resected polyps. Additionally, a polyp does not fall off after a single ligature or incomplete necrosis. This results in an increased risk of intussusception in larger polyps over time and the possibility of overlooking adenomatous neoplastic lesions. Here, we discuss two problems related to this matter and suggest corresponding countermeasures. The first problem is the inability to perform a histopathological evaluation of the entire polyp. However, small intestinal carcinoma is extremely rare in pediatric JPS. Ishida et al. reported a 3% incidence of small intestinal carcinoma in JPS, all at ≥15 years of age.^10^ Currently, conclusive evidence regarding the malignant transformation of small intestinal polyps in patients with JPS aged <15 years is lacking. Detailed endoscopic assessment is essential, and if malignancy is suspected, the treatment strategy should shift to polypectomy, in which the excised polyp can be retrieved and subjected to histopathological examination rather than EIP. Therefore, EIP should only be considered for pedunculated hamartomatous polyps that appear benign. It should also be considered in situations where conventional resection is associated with an increased risk of bleeding, such as in patients with a coagulation disorder.

The second problem is the risk of detached polyps migrating into the lower gastrointestinal tract and inducing intussusception. To address this concern, when polyps are distributed throughout the small intestine, the treatment strategy may involve initially managing distal small intestinal polyps using a trans‐anal approach, followed by treatment of proximal lesions. By treating the distal polyps first, it is expected that the risk of intussusception will be reduced, as polyps detached from the proximal small intestine are less likely to become caught on remaining distal polyps. A practical limitation of this treatment is that two DBE procedures are required.

## 4 CONCLUSION

The current case suggests that EIP is a feasible and potentially safe therapeutic option for small intestinal pedunculated polyps in a pediatric patient with JPS. It should only be used for lesions that appear clearly benign, with meticulous endoscopic assessment and close follow‐up. Additional case accumulation is crucial to establish the safety and efficacy of EIP as atherapeutic option for small intestinal polyps in children with JPS.

## CONFLICT OF INTEREST STATEMENT

The authors declare no conflicts of interest.

## ETHICS STATEMENT

Informed patient consent was obtained for publication of the case details.

## References

[1] Matsumoto T , Umeno J , Jimbo K , et al. Clinical guidelines for diagnosis and management of juvenile polyposis syndrome in children and adults‐secondary publication. J Anus Rectum Colon. 2023;7(2):115‐125. 37113581 10.23922/jarc.2023-002 PMC10129355

[2] Cohen S , Hyer W , Mas E , et al. Management of juvenile polyposis syndrome in children and adolescents: a position paper from the ESPGHAN polyposis working group. J Pediatr Gastroenterol Nutr. 2019;68(3):453‐462. 30585890 10.1097/MPG.0000000000002246

[3] Lucaciu L , Yano T , Saurin JC . Updates in the diagnosis and management of non‐ampullary small‐bowel polyposis. Best Pract Res Clin Gastroenterol. 2023;64‐65:101852. 10.1016/j.bpg.2023.101852 37652652

[4] Funayama Y , Shinozaki S , Yano T , Yamamoto H . Advancements in endoscopic management of small‐bowel polyps in Peutz‐Jeghers syndrome and familial adenomatous polyposis. Therap Adv Gastroenterol. 2023;17:17562848231218561. 38164364 10.1177/17562848231218561 PMC10757794

[5] Dofuku M , Yano T , Yokoyama K , et al. Management of pediatric Peutz‐Jeghers syndrome: highlighting the efficacy and safety of endoscopic ischemic polypectomy. J Pediatr Gastroenterol Nutr. 2025;80(3):408‐416. 39760314 10.1002/jpn3.12458

[6] Uraoka T , Takizawa K , Tanaka S , et al. Guidelines for colorectal cold polypectomy (supplement to “Guidelines for Colorectal Endoscopic Submucosal Dissection/Endoscopic Mucosal Resection”). Digest Endosc. 2022;34(4):668‐675. 10.1111/den.14250 35113465

[7] Attard TM , Cohen S , Durno C . Polyps and polyposis syndromes in children. Gastrointest Endosc Clin N Am. 2023;33(2):463‐486. 36948756 10.1016/j.giec.2022.11.001

[8] Friesen HJ , Attard TM , Liman AYJ , et al. Cold snare polypectomy in pediatric polyposis: a multicenter experience. Children (Basel, Switzerland). 2025;12(3):291. 40150574 10.3390/children12030291 PMC11940943

[9] Sakamoto H , Yamamoto H , Hayashi Y , et al. Nonsurgical management of small‐bowel polyps in Peutz‐Jeghers syndrome with extensive polypectomy by using double‐balloon endoscopy. Gastrointest Endosc. 2011;74(2):328‐333. 21704992 10.1016/j.gie.2011.04.001

[10] Ishida H , Ishibashi K , Iwama T . Malignant tumors associated with juvenile polyposis syndrome in Japan. Surg Today. 2018;48(3):253‐263. 28550623 10.1007/s00595-017-1538-2

